# TRIM27 acts as an oncogene and regulates cell proliferation and metastasis in non-small cell lung cancer through SIX3-β-catenin signaling

**DOI:** 10.18632/aging.104163

**Published:** 2020-12-02

**Authors:** Shiyuan Liu, Ying Tian, Ying Zheng, Yao Cheng, Danjie Zhang, Jiantao Jiang, Shaomin Li

**Affiliations:** 1 Department of Thoracic Surgery, The Second Affiliated Hospital of Xi'an Jiaotong University, Xi'an 710004, China; 2 Xi'an Jiaotong University Press, Xi'an 710049, China; 3 Xi'an Jiaotong University School of Medicine, Xi'an 710061, China

**Keywords:** β-catenin, lung cancer, ubiquitination, TRIM27, SIX3

## Abstract

The Wnt/β-catenin pathway plays vital roles in diverse biological processes, including cell differentiation, proliferation, migration, and insulin sensitivity. A recent study reported that the DNA-binding transcriptional factor SIX3 is essential during embryonic development in vertebrates and capable of downregulating target genes of the Wnt/β-catenin pathway in lung cancer, indicating negative regulation of Wnt/β-catenin activation. However, regulation of the SIX3-Wnt/β-catenin pathway axis remains unknown. We measured the expression of TRIM27 and SIX3 as well as investigated whether there was a correlation between them in lung cancer tissue samples. Herein, we found that the E3 ubiquitin ligase, TRIM27, ubiquitinates, and degrades SIX3. TRIM27 induces non-small cell lung cancer (NSCLC) cell proliferation and metastasis, and the expression of β-catenin, S100P, TGFB3, and MMP-9 were significantly inhibited by SIX3. Furthermore, XAV939 is a selective β-catenin-mediated transcription inhibitor that inhibited TRIM27- and SIX3-mediated NSCLC cell proliferation, migration, and invasion. Clinically, lung tissue samples of cancer patients showed increased TRIM27 expression and decreased SIX3 expression. Taken together, these data demonstrate that TRIM27 acts as an oncogene regulating cell proliferation and metastasis in NSCLC through SIX3-β-catenin signaling.

## INTRODUCTION

Lung cancer is a malignant tumor with high morbidity and mortality worldwide. According to different histopathological types, lung cancer can be divided into non-small cell lung cancer (NSCLC) and small cell lung cancer, with the former more clinically common, accounting for 80–85% of cases [1]. Lung adenocarcinoma is the most common pathological type of NSCLC and is the main type of lung cancer in women and non-smoking men. At present, the five-year survival rate of patients with lung adenocarcinoma is approximately 15–20% and a median survival time between 8–12 months; the overall survival rate is low [2]. Previous studies have shown that unlimited cell proliferation and distant metastasis of lung cancer is the leading cause of death [3–5]. Although many efforts have been made, the precise molecular mechanism of the occurrence and development of lung cancer remains unclear.

The Wnt/β-catenin pathway is one of the Wnt pathways that leads to accumulation of β-catenin in the cytoplasm, and ultimately translocation to the nucleus where combined with the TCF/LEF complex activates transcription of target genes [6, 7]. β-catenin is one of the core components of the Wnt/β-catenin signaling pathway whose expression or activity directly affects function of the signaling pathway. Moreover, Wnt/β-catenin alterations are prominent in human malignancies. Inhibition of β-catenin results in decreased expression of its downstream targets, MMP-9, C-Myc, and cyclin D1, which suppress cell cycle progression, cell proliferation, and metastasis in prostate cancer [8] and hepatocellular carcinoma [9]. The prevention of ubiquitination-mediated β-catenin degradation and promotion of β-catenin nuclear accumulation contribute to increased NSCLC cell proliferation and colony formation [10]. SIX3 is a member of the SIX family of homeoproteins that as a DNA-binding transcription factor is downregulated in lung adenocarcinoma tissues and correlated with gender, tumor size, overall survival, and recurrence of patients with lung adenocarcinoma [11]. Previous studies have shown that SIX3 acts as a suppressor in lung cancer by modulating proliferation and migration-related genes, such as S100P and TGFB3 [11], and the Wnt/β-catenin signaling pathway is involved in SIX3 function in glioma development [12] and vertebrate forebrain development [13]. Additionally, S100P promotes endometrial cancer cell proliferation by increasing nuclear translocation of β-catenin [14], and its downregulation results in gastric cancer cell apoptosis and inhibited cell colony formation by decreasing β-catenin expression [15]. In addition, ectopic activation of Wnt/β-catenin signaling contributes to upregulated TGFB3 expression in palatal epithelium and in MC3T3-E1 cells during osteoblast differentiation [16, 17]. Although these findings indicate that SIX3 may regulate tumorigenesis of lung cancer via Wnt/β-catenin signaling, the regulation of this SIX3-Wnt/β-catenin signaling axis remains largely unknown.

Tripartite motif containing 27 (TRIM27), which is an E3 ubiquitin ligase that mediates deubiquitination of RIP1, is epigenetically activated in testis [18]. Further, TRIM27 negatively regulates NOD2 and TBK1 by mediating their ubiquitination and proteasomal degradation [19, 20]. In addition, TRIM27 is a transcription repressor through interactions with myocardin related transcription factor B (MRTFB), retinoblastoma susceptibility gene (RB1), and enhancer of polycomb (EPC), and is associated with cell growth, invasion, and metastasis [21–23]. Moreover, TRIM27 is also identified as an oncogene that is highly expressed in lung, ovarian, colorectal, breast, and endometrial cancer and regulates cell cycle progression, cell proliferation, apoptosis, invasion, and metastasis [22, 24–26]. However, the underlying mechanism by which TRIM27 regulates lung cancer progression is not fully understood.

In the present study, we demonstrate that TRIM27 negatively regulates SIX3 through ubiquitination and proteasomal degradation. Further, SIX3 inhibits NSCLC cell proliferation and metastasis, whereas TRIM27 promotes NSCLC cell metastasis and proliferation in a Wnt/β-catenin-dependent manner. In addition, TRIM27 and SIX3 expression in lung cancer tissues is negatively correlated. Thus, TRIM27-SIX3-β-catenin signaling may play a key role in the regulation of cell proliferation and metastasis during NSCLC development.

## RESULTS

### SIX3 is predicted *in silico* to interact with human E3 ubiquitin ligases as a substrate

**Figure 1:**
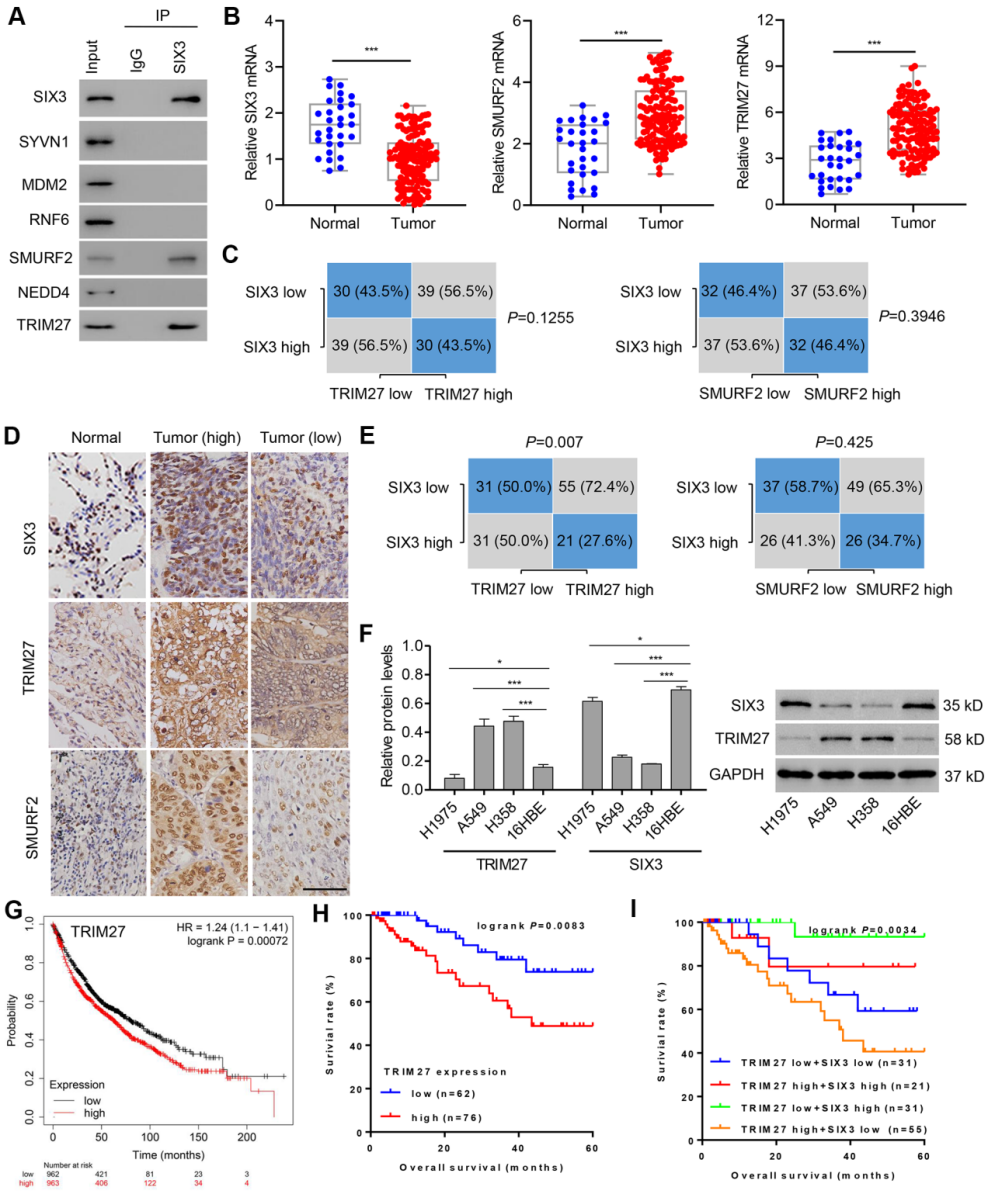
**TRIM27 and SIX3 expression levels and correlation analysis in lung cancer cells and tissues.** (**A**) A549 cell lysates underwent co-immunoprecipitation with anti-SIX3 or control IgG antibody. The immunoprecipitates were then immunoblotted with the indicated antibodies. TRIM27, SMURF2, and SIX3 expression in lung cancer tissues (n = 138) and adjacent normal lung tissues (n = 30) in our independent hospital cohort was detected by quantitative real-time PCR (**B**) and immunohistochemistry (**D**). (**C**, **E**) Correlation analysis of SIX3, SMURF2, and TRIM27 in lung cancer tissues (n = 138). Statistical analyses were performed using the Chi-square test. (**F**) TRIM27 and SIX3 expression in cells from the NSCLC cell lines H1975, A549, and H358, and the human bronchial epithelial cell line 16HBE were measured by western blot analysis. Survival probability of patients with lung cancer from a Kaplan-Meier Plotter database (**G**) and our independent hospital cohort (**H**, **I**). Scale bar: 50 μm. All experiments were repeated at least three times, and data are represented as mean ± SD. (**B**) ***P < 0.001 (Mann-Whitney U test). (**F**) *P < 0.05, ***P < 0.001 (one-way ANOVA followed by Dunnett’s test).

A previous study suggested that SIX3 inhibits the mRNA expression of metastasis- and proliferation-related genes through suppressing the activation of Wnt/β-catenin in lung cancer [11]. However, how SIX3 is regulated is unclear. To further study the regulation of the SIX3/β-catenin axis in lung cancer pathogenesis, we performed bioinformatic analysis using UbiBrowser (http://ubibrowser.ncpsb.org/), and found based on confidence level no high-confidence interactions (defined as *P* < 0.001), 10 middle-confidence interactions (0.001 ≤ *P* < 0.01), and 67 low-confidence interactions (0.01 ≤ *P* < 0.05). The significance score of each prediction is calculated as the ratio of its rank to the number of all predicted pair multiplies by the number of substrates for the corresponding E3 ubiquitin ligase. The top 20 human E3 ubiquitin ligases predicted to interact with SIX3 are shown in Supplementary Figure 1A, and the middle-confidence interactions predicted between SIX3 and human E3 ubiquitin ligases are shown in Supplementary Figure 1B. Among them, only SMURF2 and TRIM27 were predicted to interact with SIX3 (Figure 1A). We found that mRNA expression of SMURF2 and TRIM27 was increased and that of SIX3 decreased in lung cancer tissues compared with those found in adjacent normal lung tissues (Figure 1B); however, correlations between SIX3 mRNA level and SMURF2 or TRIM27 mRNA level were not significant (Figure 1C; median value used as cut-off). Similarly, the amount of protein of SMURF2 and TRIM27 was also increased and that of SIX3 was decreased in lung cancer tissues compared with adjacent normal lung tissues (Figure 1D), although we found that there was a significant correlation between the protein level of SIX3 and TRIM27, but not of SMURF2 (Figure 1E). The expression patterns of SIX3 and TRIM27 were also examined in cells from NSCLC cell lines compared with 16HBE cells (Figure 1F). SIX3 expression is associated with improved survival in lung cancer [11], whereas TRIM27 expression indicates a poor survival outcome based on a Kaplan-Meier Plotter database (Figure 1G) and our hospital cohort (Figure 1H). Importantly, we found that overall survival was highest in patients with low TRIM27 and high SIX3 expression and the shortest overall survival in those with high TRIM27 and low SIX3 expression (Figure 1I). Moreover, TRIM27 expression was associated with tumor size and lymph node metastasis, but not other clinical characteristics (Table 1).

**Table 1:** Association of TRIM27 expression with clinical characteristics of 138 patients with lung adenocarcinoma.

**Characteristics**	**Patients (n=138)**	**TRIM27 level**		***P***
		**Low (n=62)**	**High (n=76)**	
**Age**				0.057
<60	59 (42.8)	21 (33.9)	38 (50.0)	
>60	79 (57.2)	41 (66.1)	38 (50.0)	
**Gender**				0.365
Male	57 (41.3)	23 (37.1)	34 (44.7)	
Female	81 (58.7)	39 (62.9)	42 (55.3)	
**Tumor size (cm)**				0.004
<4	66 (47.8)	38 (33.9)	28 (40.8)	
>4	72 (52.2)	24 (66.1)	48 (59.2)	
**Smoking status**				0.293
Never	60 (43.5)	30 (48.4)	30 (39.5)	
Former and current smokers	78 (56.5)	32 (51.6)	46 (60.5)	
**Tumor grade**				0.053
Well and moderately differentiated	72 (52.2)	38 (61.3)	34 (44.7)	
Poorly differentiated	66 (47.8)	24 (38.7)	42 (55.3)	
**Lymph node metastasis**				0.011
Positive	85 (61.6)	31 (50.0)	54 (71.1)	
Negative	53 (38.4)	31 (50.0)	22 (28.9)	
**Disease stage**				0.165
I	26 (18.8)	16 (8.1)	10 (27.6)	
II	72 (52.2)	30 (64.5)	42 (42.1)	
III	40 (29.0)	16 (27.4)	24 (30.3)	

Differences between groups were done by the Chi-square test.

### TRIM27 ubiquitinates and degrades SIX3 in NSCLC cell lines

**Figure 2:**
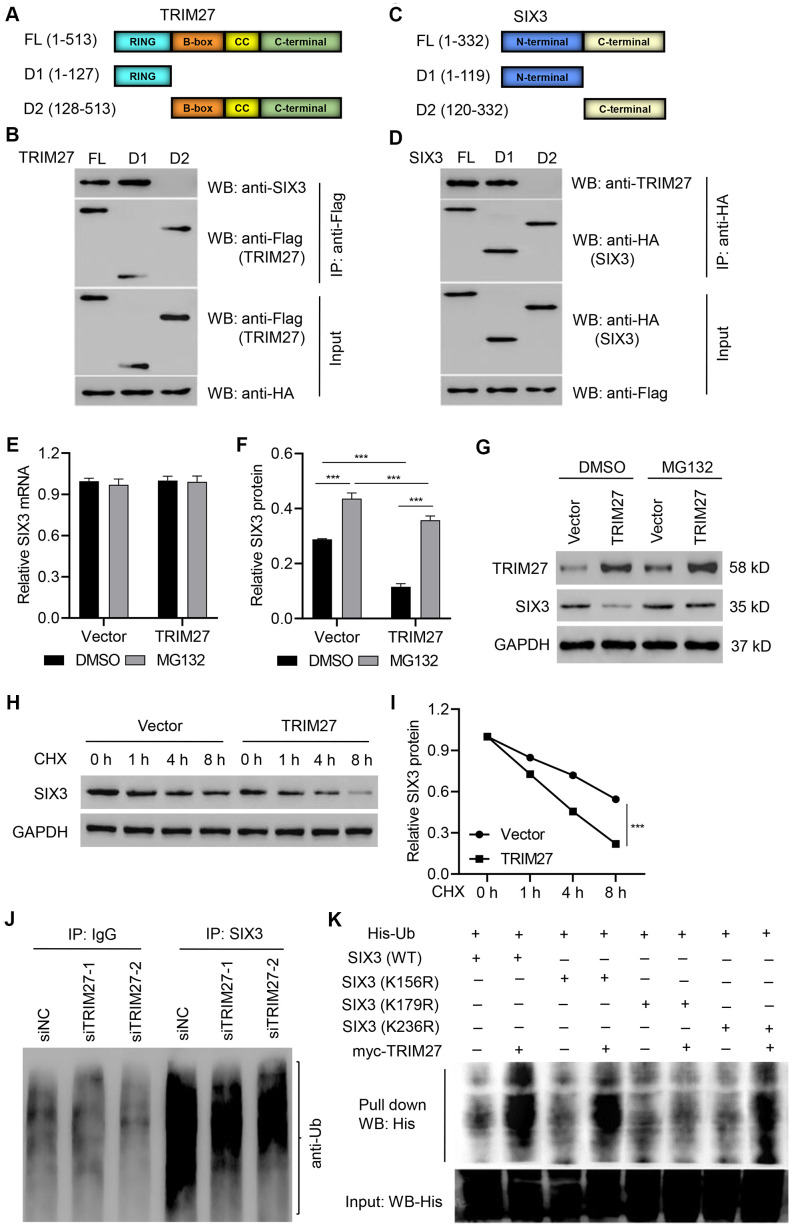
**Identification of the binding motifs involved in TRIM27-SIX3 interaction.** Schematic representation of FLAG-tagged full-length (**A**) TRIM27 or (**C**) SIX3 (FL), along with its various deletion mutants (D1 and D2). CC, coiled-coil domain. A549 cells were cotransfected with the (**B**) indicated HA-tagged SIX3 constructs along with those encoding FLAG-tagged TRIM27 or the (**D**) indicated FLAG-tagged TRIM27 constructs along with those encoding HA-tagged SIX3. Interaction between TRIM27 and SIX3 was determined by immunoprecipitation and immunoblotting. (**E**–**G**) A549 cells infected with pLVX-Puro-TRIM27 or blank pLVX-Puro vector were treated with DMSO or 10 μM MG132 for 4 h, and SIX3 expression was determined by quantitative real-time PCR and western blot analysis. (**H**, **I**) A549 cells infected with pLVX-Puro-TRIM27 or blank pLVX-Puro vector were treated with CHX (100 μg/mL), and SIX3 expression was determined by western blot analysis. (**J**) SIX3 was immunoprecipitated and immunoblotted in A549 cells transfected with siTRIM27-1 or siNC. (**K**) A549 cells were cotransfected with the SIX3 (WT) or mutant SIX3 constructs along with myc-TRIM27 and His-Ub constructs, and a pull-down assay was performed. All experiments were repeated at least three times, and data are represented as mean ± SD. (**F**) ***P < 0.001 (two-way ANOVA followed by Dunnett’s test).

TRIM27 is associated with poor prognosis of lung cancer with EGFR mutations [27], and it has been reported to regulate migration, invasion, and proliferation of lung cancer cells [25]. However, the underlying molecular mechanism is still unknown. TRIM27 consists of a RING, B-box, coiled-coil, and C-terminal domain (Figure 2A). To identify the binding domain that accounts for TIM27 binding to SIX3, we transfected expression vectors encoding FLAG-tagged TRIM27 or deletion mutants (TRIM27-D1 or TRIM27-D2) and HA-tagged SIX3 into A549 cells, followed by IP and immunoblotting with anti-FLAG or anti-SIX3 antibody. Our results showed that the RING domain on TRIM27 is necessary for interaction with SIX3 (Figure 2B). Conversely, we also generated expression constructs for HA-tagged SIX3 and a series of deletion mutants that lack different domains (Figure 2C). To map the binding motif that accounts for SIX3 binding to TRIM27, we coexpressed these constructs along with full-length FLAG-tagged TRIM27. The IP results indicate that the N-terminal of SIX3 interacts with TRIM27 (Figure 2D).

TRIM27 silencing in A549 and H358 cells significantly increased the amount of SIX3 protein compared with siNC groups, while TRIM27 overexpression in H1975 cells significantly decreased the amount of SIX3 protein compared with a blank vector group (Supplementary Figures 2, 3). Further, we found that TRIM27 overexpression in A549 cells significantly decreased the amount of SIX3 protein, an effect which was inhibited by treatment with the proteasome inhibitor MG132, with no effect on SIX3 mRNA level; a finding that indicates TRIM27 decreases SIX3 in a proteasome-dependent manner (Figure 2E–2G). To further establish that TRIM27 regulates SIX3 stability, we treated cells with CHX and determined the half-life of SIX3. As shown in Figure 2H, 2I, SIX3 stability was dramatically decreased in A549 cells overexpressing TRIM27. These results demonstrate that TRIM27 destabilizes SIX3 in A549 cells. We then investigated whether TRIM27 affects the ubiquitination of SIX3 in A549 cells. As shown in Figure 2J, TRIM27 silencing in A549 cells resulted in the inhibition of SIX3 ubiquitination compared with that found in the siNC group, suggesting TRIM27 ubiquitinates SIX3. Furthermore, our pull-down results indicate that SIX3 K179 is required for TRIM27-induced ubiquitination of SIX3 (Figure 2K).

### SIX3 regulates NSCLC cell proliferation, invasion, and migration

**Figure 3:**
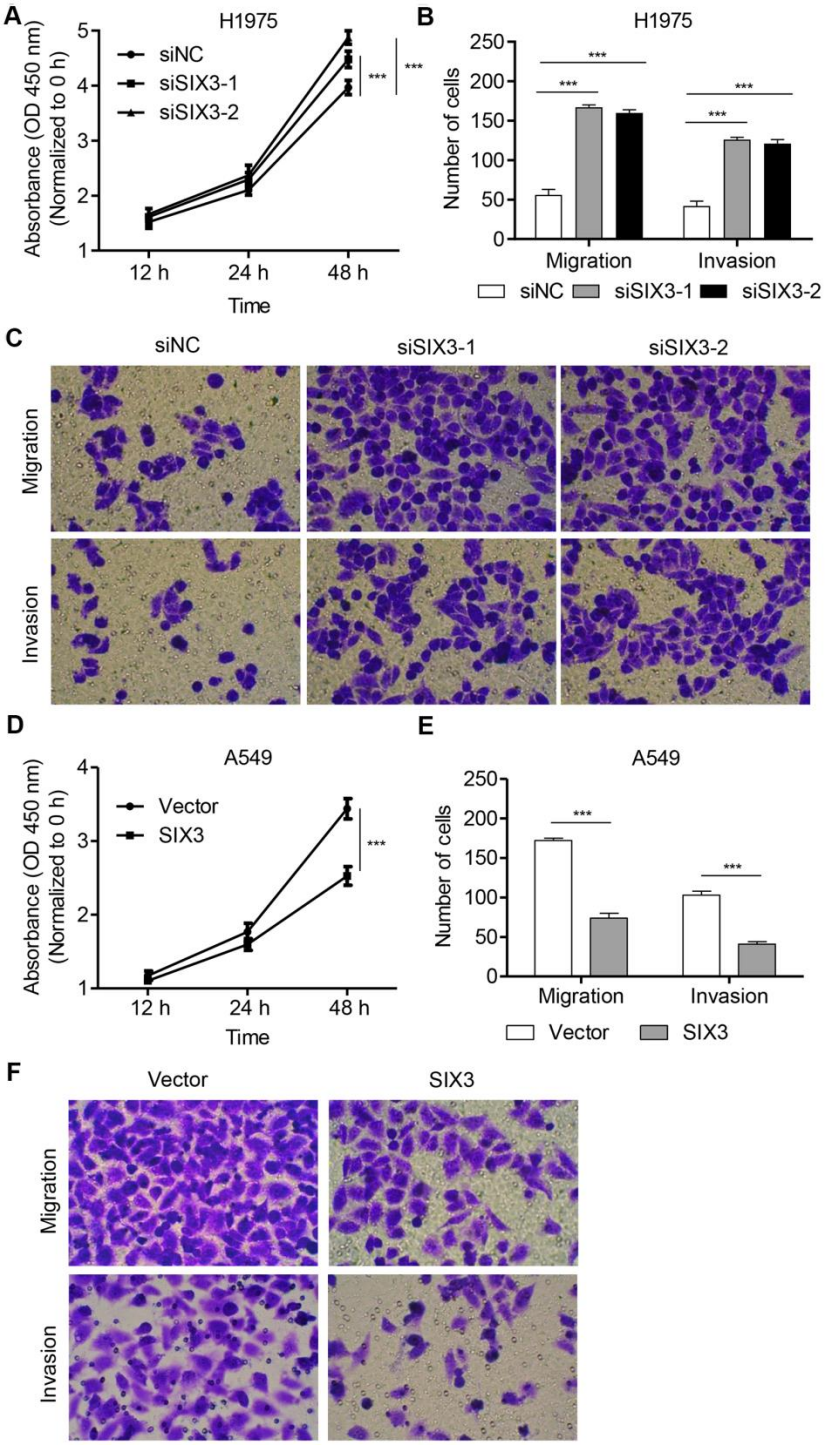
**SIX3 regulates NSCLC cell proliferation, invasion, and migration.** H1975 cells were transfected with siSIX3-1, siSIX3-2, or siNC, and cell proliferation (**A**), migration (**B**), and invasion (**C**) were determined by CCK-8 and transwell assay. A549 cells were infected with pLVX-Puro-SIX3 or blank pLVX-Puro vector, and cell proliferation (**D**), migration (**E**), and invasion (**F**) were determined by CCK-8 and transwell assay. All experiments were repeated at least three times, and data are represented as mean ± SD. ***P < 0.001 (two-way ANOVA followed by Dunnett’s test).

To examine the roles of SIX3 in the regulation of NSCLC cell phenotypes, H1975 cells were transfected with SIX3 siRNA while A549 cells were infected with a TRIM27-expressing lentivirus infection. Quantitative real-time PCR as well as western blot analysis demonstrated decreased levels of SIX3 in siRNA-transfected H1975 cells compared with the siNC group, with the lowest levels detected in cells with siRNA-1 and siRNA-2 transfection (Supplementary Figure 2E). These two siRNAs were therefore used for subsequent experiments. A549 cells with SIX3 overexpression showed significantly increased expression of SIX3 compared with that found in the blank vector group (Supplementary Figure 2F). We also found that proliferation, invasion, and migration of H1975 cells transfected with SIX3-siRNA were markedly increased compared with cells transfected with siNC (Figure 3A-C). Furthermore, the opposite phenotypes of A549 cells infected with SIX3 expressing lentivirus were observed compared with cells infected with a blank vector (Figure 3D–2F). These findings demonstrate the anti-proliferation and anti-motility function of SIX3 in NSCLC cells, indicating that SIX3 may play an important role in NSCLC as a novel suppressor.

### SIX3 inhibits NSCLC cell proliferation, invasion, and migration induced by TRIM27

**Figure 4:**
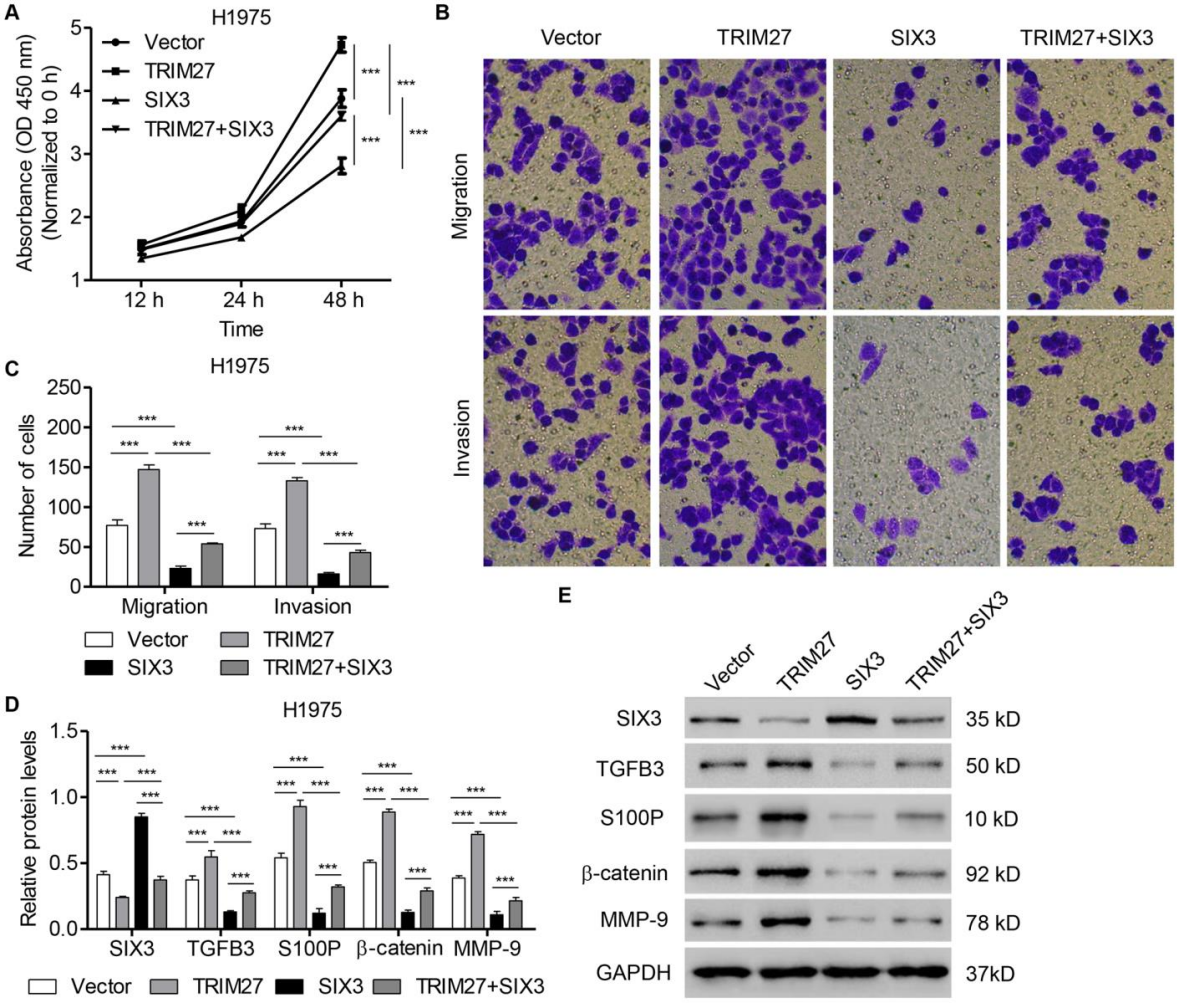
**SIX3 overexpression inhibits NSCLC cell proliferation, migration, and invasion induced by TRIM27 overexpression.** H1975 cells were infected with blank pLVX-Puro vector, pLVX-Puro-TRIM27, or pLVX-Puro-SIX3, and cell proliferation (**A**), migration (**B**), invasion (**C**), and related protein expression (**D**, **E**) were determined by CCK-8, transwell assay, and western blot analysis. All experiments were repeated at least three times, and data are represented as mean ± SD. ***P < 0.001 (two-way ANOVA followed by Dunnett’s test).

To further determine the roles of SIX3 and TRIM27 in NSCLC cell lines, H1975 cells were infected with SIX3- and/or TRIM27-expressing lentivirus. As shown in Figure 4A–4C, TRIM27 overexpression in H1975 cells induced cell proliferation, migration, and invasion, all of which were significantly inhibited by SIX3 overexpression. We next examined whether TRIM27 regulates Wnt/β-catenin signaling in a SIX3-dependent manner. Our western blot analysis revealed that TRIM27 overexpression inhibited SIX3, but increased β-catenin, S100P, TGFB3, and MMP-9 expression (Figure 4D, 4E). However, we found that overexpression of SIX3 in TRIM27-overexpressing cells reversed the effect of TRIM27 overexpression on the expression of these proteins, suggesting that TRIM27 regulates Wnt/β-catenin signaling in a SIX3-dependent manner.

**Figure 5:**
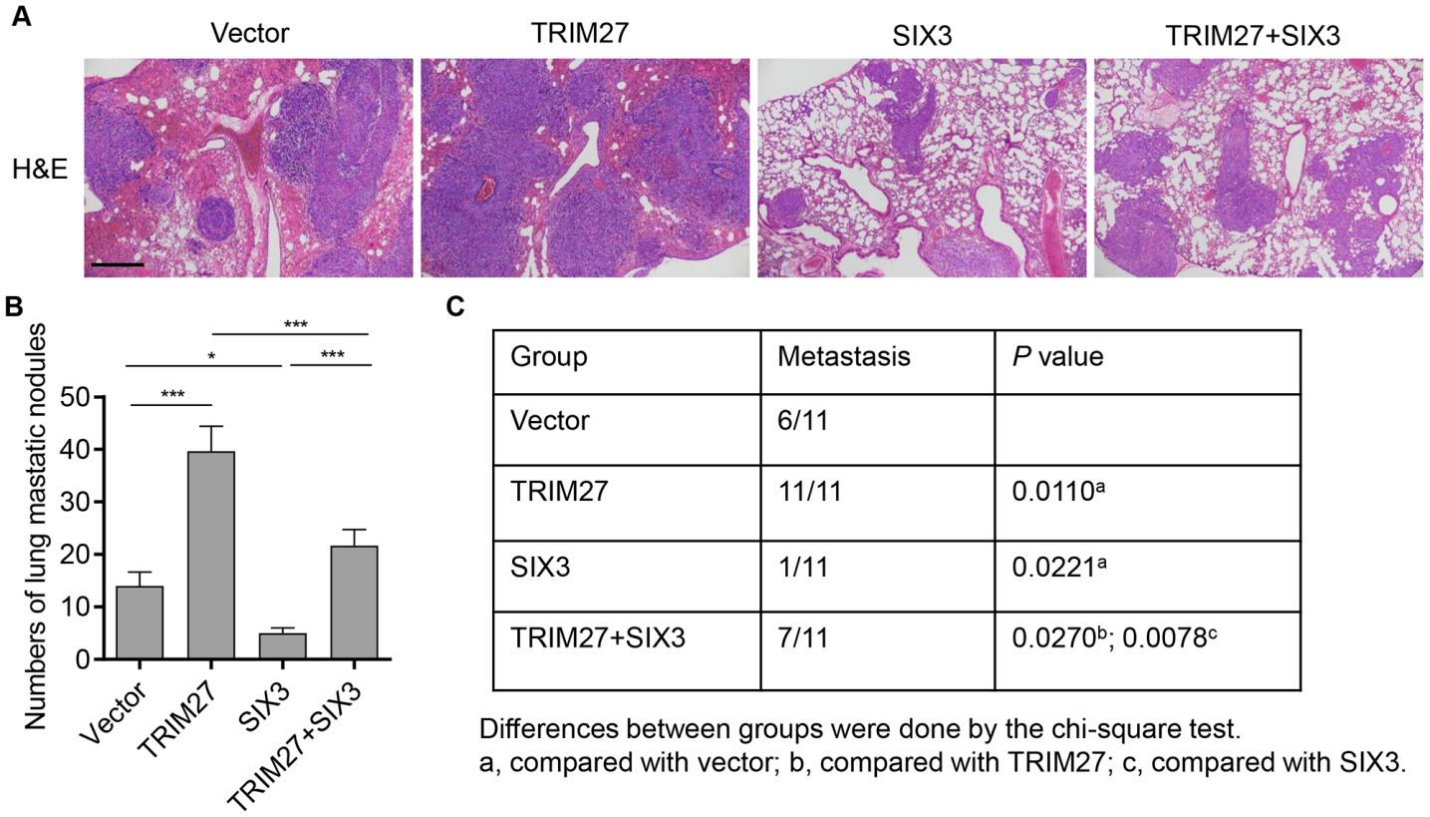
**The roles of TRIM27 and SIX3 on lung metastasis *in vivo*.** (**A**) Histology of single lung lobe from mice intravenously injected with A549 cells stably infected with blank pLVX-Puro vector, pLVX-Puro-TRIM27, or pLVX-Puro-SIX3. Scale bars: 500 μm. (**B**) Quantification of microscopic nodules in the lungs of each group. (**C**) The incidence of metastasis in mice after intravenous tail injection of each cell type is shown in the table. All experiments were repeated at least three times, and data are represented as mean ± SD. *P < 0.05, ***P < 0.001 (two-way ANOVA followed by Dunnett’s test).

To investigate the biological function of TRIM27 and SIX3 in NSCLC cells *in vivo*, TRIM27- and/or SIX3-overexpressing A549 cells were intravenously injected through the tail vein into nude mice and cell metastasis was monitored for 3 weeks. Mice bearing TRIM27-overexpressing A549 cells showed increased cell metastasis compared with mice injected with blank vector infected cells, an effect which was inhibited by SIX3 overexpression (Figure 5A). We also found that the number of lung metastasis nodules and the incidence of metastasis in mice were significantly increased in the TRIM27 group, which was inhibited by SIX3 overexpression (Figure 5B, 5C). Taken together, our results indicate that SIX3 is a negative regulator of TRIM27-induced metastasis.

### TRIM27 and SIX3 regulate NSCLC cell migration and invasion partly through the Wnt/β-catenin pathway

**Figure 6:**
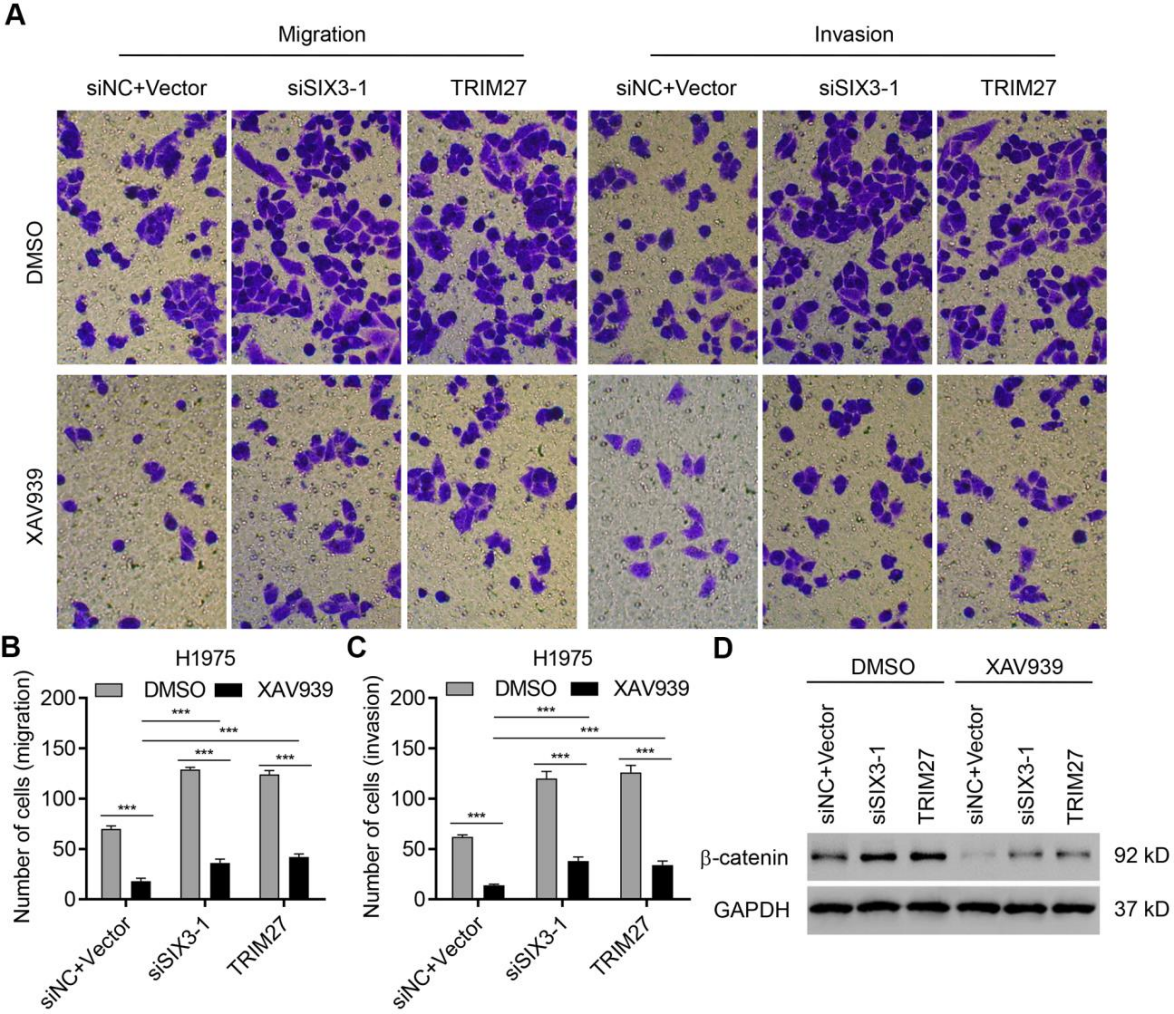
**TRIM27 and SIX3 regulate NSCLC cell migration and invasion partly through the Wnt/β-catenin pathway.** H1975 cells transfected or infected with siSIX3-1, pLVX-Puro-TRIM27, or both siNC and blank pLVX-Puro vector were treated with DMSO or 10 μM XAV939, and cell migration, invasion, and related protein expression were determined by transwell assay (**A**–**C**) and western blotting (**D**). All experiments were repeated at least three times, and data are represented as mean ± SD. ***P < 0.001 (two-way ANOVA followed by Dunnett’s test).

To confirm the role of Wnt/β-catenin signaling in TRIM27- and SIX3-mediated NSCLC cell migration and invasion, the selective β-catenin-mediated transcription inhibitor XAV939 was used. As shown in Figure 6A–6D, SIX3 silencing and TRIM27 overexpression in H1975 cells significantly increased β-catenin expression as well as cell invasion and migration, which were partly suppressed by the addition of XAV939. These findings suggest that TRIM27 and SIX3 regulate NSCLC cell migration and invasion partly through the Wnt/β-catenin pathway.

## DISCUSSION

Targeted molecular therapy improves the outcomes of patients with NSCLC. For instance, EGFR, ALK, and ROS1 tyrosine kinase inhibitors against tumors with EGFR mutations, ALK fusions, and ROS1 fusions respectively, are already in use as standard treatments in lung cancer in clinical settings [28]. Other molecular alterations, such as members of the SIX family, also regulate cell proliferation, apoptosis, and metastasis in lung cancer [29]. Accordingly, exploring new molecular markers is conducive to precision treatment. In the present study, we discovered that ubiquitination and proteasomal degradation of SIX3 by TRIM27, an E3 ubiquitin ligase, in NSCLC promotes cell proliferation and metastasis partly through the Wnt/β-catenin pathway. Furthermore, we found that TRIM27 and SIX3 expression in lung cancer tissues is negatively correlated.

As ubiquitination is associated with many biological processes, such as cell differentiation, proliferation, apoptosis, cell cycle, DNA repair, and inflammation [30], SIX3, a tumor suppressor in lung cancer, was bioinformatically predicted as a substrate for several human E3 ubiquitin ligases. Among them, SMURF1, MDM2, SMURF2, TRIM27, and NEDD4L are involved in lung cancer progression. SMURF1 regulates lung cancer cell growth and migration through ubiquitination of PIPKIγ [31], and inhibition of the proteasome-mediated degradation of MDM2 promotes NSCLC cell proliferation though p53 signaling [32]. Similarly, SMURF2-dependent ubiquitin degradation of TGFβ receptors contributes to the inhibition of lung cancer cell proliferation, migration, and invasion [33], whereas low NEDD4L expression correlates with poor outcome in patients with lung cancer and promotes TGFβ-induced epithelial-to-mesenchymal transition [34]. Although it is known that TRIM27 is upregulated in lung cancer tissues, and its silencing significantly inhibits lung cancer cell migration, adhesion, invasion, and proliferation [25], the underlying molecular mechanism is still not understood. TRIM27 is a novel E3 ubiquitin ligase that ubiquitinates PTEN, which leads to decreased PTEN phosphatase activity [35]. We confirmed that TRIM27 interacts with and ubiquitinates SIX3; however, TRIM27 as a transcription repressor has been found to regulate integrin β1 expression by altering its mRNA stability and the rate of translation via MRTF-B [21], which is inconsistent with our findings that TRIM27 only regulates the translation of SIX3 translation, but not its transcription. TRIM27 also regulates gene transcription through interactions with EPC, which has dual transcriptional functions [23]. These findings suggest that the repressive and transactivating activities of TRIM27 are dependent on the transcriptional functions of the complexes interacting with it. Moreover, SIX3 was downregulated in cells from lung cancer cell lines and inhibited NSCLC cell proliferation and metastasis, findings which are consistent with those of previous studies [11, 12]. Importantly, SIX3 inhibited TRIM27-induced NSCLC cell proliferation and metastasis indicating the involvement of SIX3 in TRIM27-mediated tumorigenesis of lung cancer.

TRIM27 and SIX3 mediate the expression of S100P, β-catenin, TGFB3, and MMP-9 through β-catenin signaling. In agreement with our findings, SIX3 inhibits NSCLC cell phenotypes by inhibiting S100P as well as TGFB3 expression through suppression of Wnt/β-catenin signaling [11]. Similarly, inhibition of Wnt/β-catenin signaling or expression of its downstream target MMP-9, results in decreased cell proliferation and metastasis in breast cancer [7] and hepatocellular carcinoma [9]. Thus, these findings suggest that TRIM27 and SIX3 regulate NSCLC cell metastasis and proliferation through Wnt/β-catenin signaling. Previous studies have also reported that S100P promotes cell migration and invasion in lung cancer [36], and that it regulates proliferation, apoptosis, and colony formation by mediating the nuclear translocation and expression of β-catenin in endometrial and gastric cancer [14, 15], suggesting that S100P similarly regulates lung cancer through the Wnt/β-catenin pathway. However, the exact mechanism how SIX3 and TRIM27 regulate S100P in lung cancer is not known, and thus, warrants further investigation. In the present study, our results showed that the invasion/migration ability of cells either overexpressing TRIM27 or siSIX3 in Wnt/β-catenin inhibitor conditions remain higher compared with control cells treated with a Wnt/β-catenin inhibitor, suggesting that TRIM27/SIX3 regulate cell invasion and migration through not only the Wnt/β-catenin pathway, but also other signaling pathways. Therefore, the molecular mechanism by which TRIM27/SIX3 regulates NSCLC cell invasion and migration are not fully elucidated and subject to further investigation.

Previous studies have provided evidence that SIX3 mRNA expression is upregulated in NSCLC and lung adenocarcinoma tissues compared with that of normal lung tissue [29], a finding which is inconsistent with our decreased SIX3 protein levels in lung cancer tissues measured by IHC and western blotting. However, SIX3 is downregulated in lung cancer tissues compared with adjacent normal lung tissues measured by IHC analysis [11], which is in line with our results. In addition, we found that TRIM27 is upregulated in lung cancer tissues compared with normal lung tissues in our cohort, which is consistent with that found in a previous study [25], and that higher TRIM27 expression correlates with lower SIX3 expression in lung cancer tissue samples from our cohort. This evidence strongly support our hypothesis and clarifies a relationship between SIX3 and TRIM27 in the pathogenesis of lung cancer. A previous study also found that SIX3 expression is associated with tumor size, gender, survival, and recurrence in lung cancer, suggesting that SIX3 may be an informative prognostic marker for lung cancer [11]. TRIM27 expression is associated with poor prognosis of EGFR-mutated lung cancers, indicating that TRIM27 status may be related to anticancer therapy response in lung cancer with EGFR mutations [27]. However, the prognostic import of either TRIM27 or SIX3 for lung cancer of different tumor stage or histology requires further investigation.

In conclusion, our results provide the first evidence that TRIM27 acts as an oncogene regulating cell proliferation and metastasis in NSCLC through SIX3-β-catenin signaling, which is targeted to SIX3 by ubiquitination.

## MATERIALS AND METHODS

### Patients and tissue samples

Primary tumor samples were collected from 138 patients at The Second Affiliated Hospital of Xi'an Jiaotong University who underwent surgery for lung cancer between 2011 and 2014. This study protocol was approved by the Ethics Committee of The Second Affiliated Hospital of Xi'an Jiaotong University and written informed consent was provided prior to patient enrollment. Lung cancer and adjacent normal tissues were immediately flash frozen in liquid nitrogen following collection and used for quantitative real-time PCR analysis, otherwise formalin-fixed paraffin-embedded lung cancer specimens underwent immunohistochemistry (IHC) analysis using SMURF2 rabbit polyclonal antibody (Abcam; ab272897), TRIM27 rabbit polyclonal antibody (Abcam; ab78393), or SIX3 rabbit polyclonal antibody (Bioss Biotechnology Company, Beijing, China; bs-11970R). Immunoreactivity was scored by two investigators based on the percentage of positively stained cells and staining intensity, which ranged from 0 to 3. Our cohort of 138 patients with lung cancer was parsed into two subgroups by IHC staining comprised of a low-expression group with an IHC score less than 50% and a high-expression group with an IHC score over 50%.

### Cell culture

Human NSCLC cell lines H1975, A549, and H358, and human bronchial epithelial cell line 16HBE were purchased from American Type Culture Collection (ATCC, Manassas, VA, USA). All cell lines were cultured in RMPI-1640 medium (Thermo Fisher Scientific, Rockford, IL, USA) containing 10% fetal bovine serum (FBS; Gibco BRL Ltd, Paisley, Scotland) and antibiotics (100 U/mL penicillin and 100 mg/L streptomycin; Mediatech, Inc., Herndon, VA, USA), and were incubated in a humidified atmosphere containing 5% carbon dioxide at 37° C.

### Cell transfection

A549, H358, and H1975 cells were transfected with Lipofectamine 2000 transfection reagent (Invitrogen Life Technologies, Carlsbad, CA, USA) using small interfering RNA (siRNA) to knockdown TRIM27 or SIX3, following the manufacturer’s instructions; scrambled siRNA was used as a negative control (siNC; Thermo Fisher Scientific). The following siRNA sequences were used for knockdown: human TRIM27 (siTRIM27-1, 5'-CCCAGUUCUCUUGCAACAU-3'; siTRIM27-2, 5'-GGGCUGAAAGAAUCAGGAU-3'; and siTRIM27-3, 5'-GGAUUCUGGGCAGUGUCUU-3') and human SIX3 (siSIX3-1, 5'-UCAACAAACACGAGUCGAU-3'; siSIX3-2, 5'-UCCUUGAGAACCACAAGUU-3'; and siSIX3-3, 5'-GACUCGGAAUGUGAUGUAU-3'). The *TRIM27* and *SIX3* coding region was subcloned into the pLVX-Puro lentiviral vector (Clontech, Mountain View, CA, USA); blank pLVX-Puro lentiviral vector was used as a negative control. To generate high-titer lentivirus, vectors encoding the target gene and packaging plasmids were cotransfected into HEK 293T cells for 4–6 h using Lipofectamine 2000 transfection reagent (Invitrogen Life Technologies) according to the manufacturer’s instructions. Forty-eight hours after transfection, viral particles in cell culture medium were collected and infected A549 and H1975 cells.

Full-length *TRIM27* or *SIX3* and their truncated mutant complementary DNAs (cDNAs) were cloned and inserted into a pCMV-FLAG vector, and generated plasmids were designated as TRIM27 (or SIX3)-FL, TRIM27 (or SIX3)-D1, and TRIM27 (or SIX3)-D2. A549 cells were cotransfected with the indicated HA-tagged SIX3 or FLAG-tagged TRIM27 constructs along with those encoding FLAG-tagged TRIM27 or HA-tagged SIX3 using Lipofectamine 2000 transfection reagent according to the manufacturer's instructions.

Further, full-length *SIX3* and truncated mutant cDNAs were cloned and inserted into a pCMV-Tag 2B vector, and generated plasmids were designated as SIX3 (WT), SIX3 (K156R), SIX3 (K179R), and SIX3 (K236R). Mutations were introduced into SIX3 with the QuikChange II Site-directed Mutagenesis kit (Agilent Technologies, Santa Clara, CA, USA). Myc-tagged TRIM27 sequence was purchased from GENEWIZ, lnc. (Suzhou, China) and cloned into a p-DONR221 vector to express myc-TRIM27. For his-ubiquitin (Ub), human ubiquitin was cloned into a pcDNA-DEST40 vector with a His tag. All constructs and mutants were confirmed by sequencing. A549 cells were cotransfected with the SIX3 (WT) or mutant SIX3 constructs along with the myc-TRIM27 and His-Ub constructs using Lipofectamine 2000 transfection reagent according to the manufacturer's instructions.

### Protein stability

To evaluate protein stability, A549 cells infected with pLVX-Puro-TRIM27 or blank pLVX-Puro vector were treated with 100 μg/mL cycloheximide (CHX; Merck Millipore, Germany) during indicated times and harvested. Protein quantity of SIX3 was then determined by western blot analysis.

### Co-immunoprecipitation and ubiquitination assay

The details of these procedures were described previously [37]. Anti-SIX3 (Santa Cruz Biotechnology, Dallas, TX, USA; sc-81985), anti-FLAG (Abcam, Cambridge, MA, USA; ab205606), anti-HA (Abcam; ab9110), and anti-IgG (Santa Cruz Biotechnology; sc-2027) antibodies were used for co-immunoprecipitation (Co-IP). Immunoprecipitates were washed at least five times and subjected to western blot analysis using anti-TRIM27 (Abcam; ab78393), anti-NEDD4 (Abcam; ab236512), anti-SMURF2 (Abcam; ab94483), anti-RNF6 (Abcam; ab204506), anti-SYVN1 (Abcam; ab170901), anti-MDM2 (Abcam; ab16895), and anti-SIX3 (Abcam; ab172131) antibodies. For ubiquitination assays, the lysates of A549 cells transfected with siTRIM27-1 or siNC were used for IP with an anti-IgG (Santa Cruz Biotechnology; sc-2027) or anti-SIX3 antibody (Santa Cruz Biotechnology; sc-81985) and Protein A/G PLUS-Agarose (Novex, Oslo, Norway), which was performed at 4° C overnight. The eluted proteins were then detected by western blot analysis using an anti-ubiquitin (Ub) antibody (Abcam, ab7780).

### His-ubiquitin pull-down assay

A549 cells were cotransfected with SIX3 (WT) or mutant SIX3 constructs along with myc-TRIM27 and His-Ub constructs. After 48 h transfection, cells lysates were incubated with Ni^2+^-NTA agarose beads (Qiagen, Hilden, Germany). The washed complexes were then eluted by boiling in sodium dodecyl sulfate (SDS) sample buffer, separated by SDS-polyacrylamide gel electrophoresis (SDS-PAGE), and analyzed by western blotting.

### Quantitative real-time PCR assay

RNA was harvested from human lung cancer cell lines using the RNeasy Mini Kit (Qiagen). RNA (1 μg) was isolated per sample and cDNA was prepared for quantitative real-time PCR using the High Capacity cDNA Reverse transcription kit (Applied Biosystems, Foster, CA, USA). The specific primers for quantitative real-time PCR were as follows: SMURF2-F: 5'-AGAACTACGCAATGGGAGC-3'; SMURF2-R: 5'-TAGCCTTCTGGTAGGTCTGG-3'; TRIM27-F: 5'-AGGACCTGCCTGACAACC-3'; TRIM27-R: 5'-AGGACCTGCCTGACAACC-3'; SIX3-F: 5'-CAAACTTCGCCGATTCTCACC-3'; SIX3-R: 5'-TCGATGTCGCCCGTCTCCTC-3'; GAPDH-F: 5'-AATCCCATCACCATCTTC-3'; and GAPDH-R: 5'-AGGCTGTTGTCATACTTC-3'. Relative mRNA expression level of the gene was normalized to that of GAPDH in the same sample.

### Western blot assay

Cell lysates were prepared in radioimmunoprecipitation assay buffer. Proteins were then dissolved in SDS-PAGE, transferred onto nitrocellulose membranes (GE Healthcare, Little Chalfont, UK), and incubated with anti-TRIM27 (Abcam; ab78393), anti-SIX3 (Abcam; ab172131), anti-β-catenin (Cell Signaling Technology, Danvers, MA, USA; #8480), anti-TGFB3 (Abcam; ab15537), anti-S100P (Abcam; ab133554), anti-MMP-9 (Abcam; ab73734), anti-His (Abcam; ab9108), and anti-GAPDH antibody (Cell Signaling Technology; #5174) at 4° C overnight, and incubated with the respective secondary antibody (Beyotime Institute of Biotechnology, Haimen, China; A0208 and A0216) at 25° C for 1 h. Protein bands were visualized using an enhanced chemiluminescence detection system and identified by a BioSpectrum Imaging System (UVP LLC, Upland, CA, USA).

### Cell proliferation assay

Cell proliferation was determined by the cell counting kit-8 (CCK-8) assay. Exponentially growing cells were seeded in 96-well plates with 3,000 cells per well and cultured overnight. A549 cells were subsequently infected with pLVX-Puro-SIX3 or blank vector and H1975 cells were transfected with siSIX3 or siNC, otherwise H1975 cells were infected with pLVX-Puro-SIX3, pLVX-Puro-TRIM27, or blank vector, following culture overnight. At 12, 24, and 48 h, CCK-8 solution was added to each well and incubated for 1 h at 37° C. Cell proliferation was determined according to the optical density measured at a wavelength of 450 nm.

### Transwell assay

Exponentially growing cells were seeded in 6-well plates with 300,000 cells per well and cultured overnight. A549 cells were subsequently infected with pLVX-Puro-SIX3 or blank vector, and H1975 cells were transfected with siSIX3 or siNC in the presence or absence of XAV939 (10 μM) or dimethyl sulfoxide (DMSO), otherwise H1975 cells were infected with pLVX-Puro-SIX3, pLVX-Puro-TRIM27, or blank vector in the presence or absence of XAV939 (10 μM) or DMSO, following culture overnight. Cells were then seeded in serum-free growth medium and incubated for one day. The upper transwell chamber (Costar, Cambridge, MA, USA) precoated with or without Matrigel (Becton Dickinson, Oxford, UK) was filled with cells resuspended in 300 μL RMPI-1640 containing 10% FBS with a density of 60,000 cells per well. An aliquot (700 μL) of RMPI-1640 containing 10% FBS was added to the lower chamber. After 24-h incubation, cells in the lower chamber were fixed with 4% paraformaldehyde and stained with crystal violet for 30 min. Non-migrated cells were removed with a cotton swab. Cell number was determined using a XDS-500D inverted microscope (Shanghai Caikon Optical Instrument Co., Ltd., Shanghai, China).

### Animal experiments

A total of 1 × 10^6^ A549 cells transduced with pLVX-Puro-TRIM27, pLVX-Puro-SIX3, or blank vector were harvested, resuspended in 100 μL phosphate-buffered saline (PBS), and then intravenously injected through the tail vein into 5–6-week-old female BALB/c nude mice (weight: 16–18 g; 11 mice per group). Three weeks after injection, the mice were sacrificed and lung tissue samples were collected from the xenograft mice and fixed in 10% Bouin’s solution for 24 h. The number of lung tumor nodules was assessed under a dissecting microscope (Carl Zeiss GmbH, Oberkochen, Germany) by two researchers and blindly checked by each other. Furthermore, lungs were washed in PBS, fixed in 4% formalin solution for 48 h, and then dehydrated, embedded, and cryosectioned for hematoxylin and eosin staining. Animal experiments were approved by the institutional ethical committee of The Second Affiliated Hospital of Xi'an Jiaotong University and performed according to legal requirements.

### Statistics

All experiments were run in triplicate. The results are presented in the form of mean ± standard deviation (SD). Data were analyzed with GraphPad Prism (version 8.0.2; GraphPad Software Inc, San Diego, CA, USA). Statistical analyses were performed using the Mann-Whitney U test or ANOVA. Differences were accepted as statistically significant with a *P*-value < 0.05.

## Supplementary Material

Supplementary Figures
