# Supplementary material for: TRIM27 acts as an oncogene and regulates cell proliferation and metastasis in non-small cell lung cancer through SIX3-β-catenin signaling
Source: Aging (Albany NY). 2020 Dec 2;12(24):25564–80. doi: 10.18632/aging.104163 (PMC7803540; doi:10.18632/aging.104163)
Supplement: Supplementary Figures [file aging-12-104163-s001.pdf]

SUPPLEMENTARY FIGURES

A

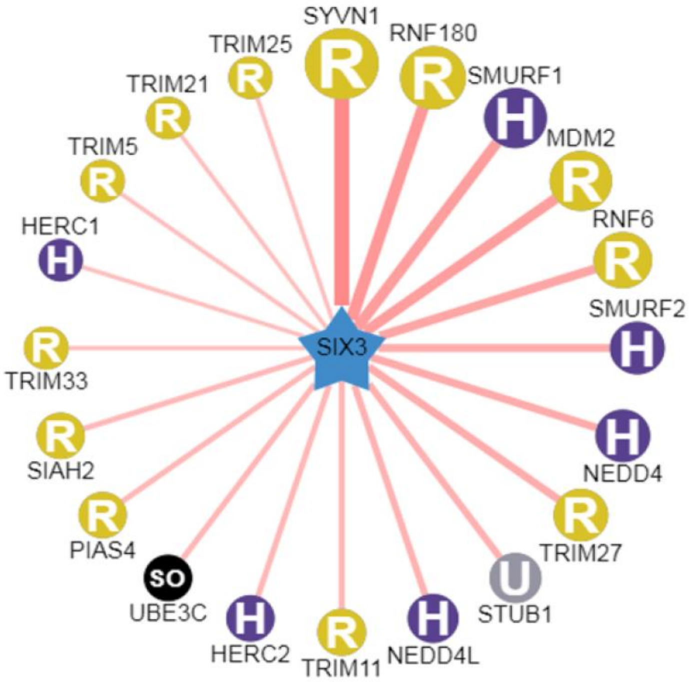

B

Predicated E3 ligases	RecName	Score
SYVN1	E3 ubiquitin-protein ligase synoviolin	0.746
RNF180	E3 ubiquitin-protein ligase RNF180	0.719
SMURF1	E3 ubiquitin-protein ligase SMURF1	0.709
MDM2	E3 ubiquitin-protein ligase Mdm2	0.707
RNF6	E3 ubiquitin-protein ligase RNF6	0.694
SMURF2	E3 ubiquitin-protein ligase SMURF2	0.682
NEDD4	E3 ubiquitin-protein ligase NEDD4	0.682
TRIM27	Zinc finger protein RFP	0.681
STUB1	E3 ubiquitin-protein ligase CHIP	0.672
NEDD4L	E3 ubiquitin-protein ligase NEDD4-like	0.669

Supplementary Figure 1. SIX3 is bioinformatically predicted to be a substrate interacted with human E3 ubiquitin ligases. (**A**) Top 20 predicated human E3 ubiquitin ligases interact with SIX3. (**B**) Middle confidence interaction ($0.001 \leq P < 0.01$) between SIX3 and human E3 ubiquitin ligases.

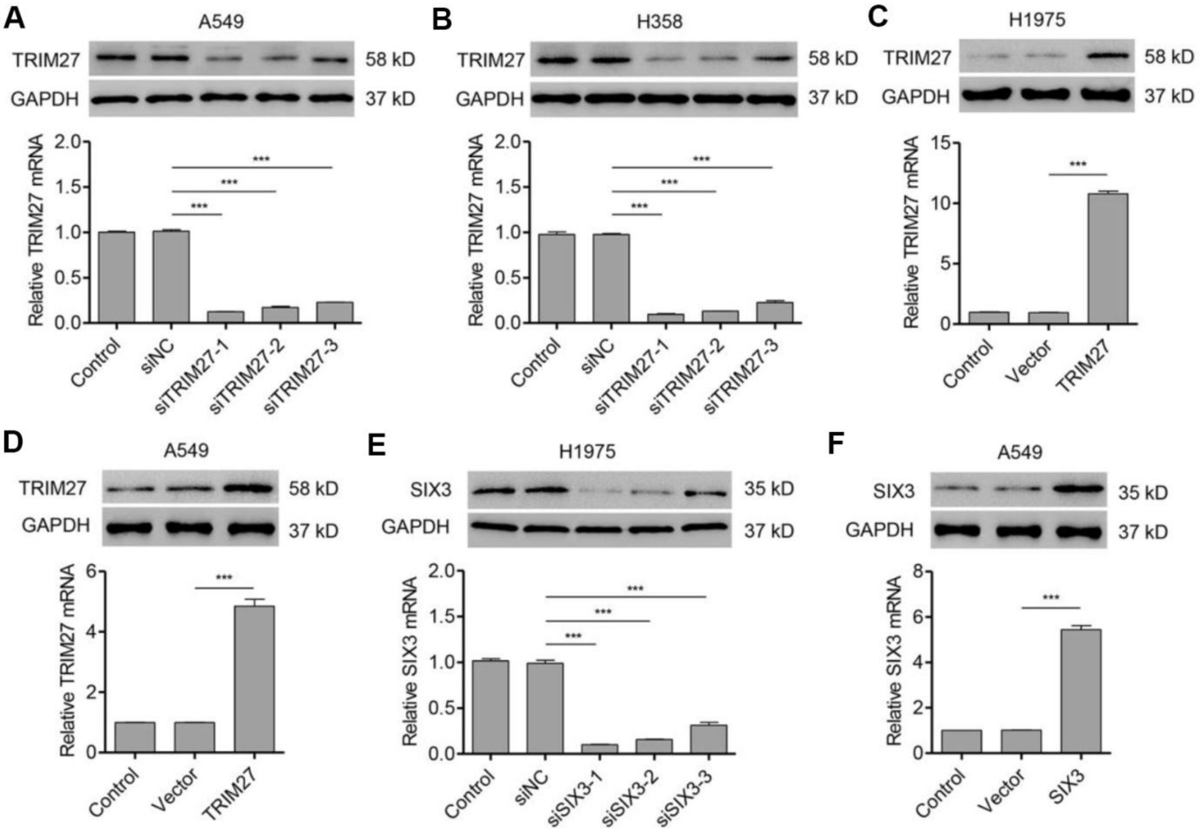

Supplementary Figure 2. TRIM27 and SIX3 expression in NSCLC cell lines. (**A**, **B**) A549 and H358 cells were transfected with siTRIM27-1, siTRIM27-2, siTRIM27-3, or siNC, and the expression of TRIM27 was measured by western blotting. (**C**, **D**) H1975 and A549 cells transfected with blank pLVX-Puro vector or pLVX-Puro-TRIM27, and the expression of TRIM27 was measured by western blotting. (**E**, **F**) H1975 cells were transfected with siSIX3-1, siSIX3-2, siSIX3-3, or siNC while A549 cells were transfected with blank pLVX-Puro vector or pLVX-Puro-SIX3, and the expression of SIX3 was measured by western blotting. All experiments were repeated at least three times, and data are represented as mean ± SD. ****P* < 0.001 (one-way ANOVA followed by Dunnett's test).

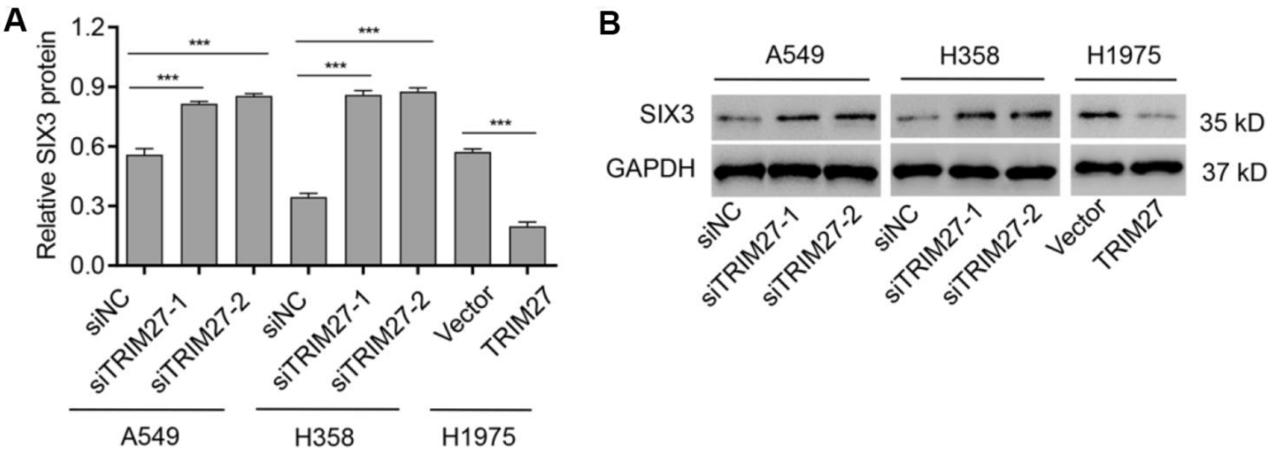

Supplementary Figure 3. SIX3 expression in NSCLC cell lines under TRIM27 knockdown or overexpression. (**A**, **B**) H358 and A549 cells were transfected with siTRIM27-1, siTRIM27-2, or siNC while H1975 cells were infected with pLVX-Puro-TRIM27 or blank pLVX-Puro vector, and the SIX3 expression levels were determined by western blotting. All experiments were repeated at least three times, and data are represented as mean ± SD. ****P* < 0.001 (one-way ANOVA followed by Dunnett's test).
